# A highly annotated database of genes associated with platinum resistance in cancer

**DOI:** 10.1038/s41388-021-02055-2

**Published:** 2021-10-13

**Authors:** Dongqing Huang, Sara R. Savage, Anna P. Calinawan, Chenwei Lin, Bing Zhang, Pei Wang, Timothy K. Starr, Michael J. Birrer, Amanda G. Paulovich

**Affiliations:** 1 Clinical Research Division, Fred Hutchinson Cancer Research Center, Seattle, WA USA; 2 Lester and Sue Smith Breast Center, Baylor College of Medicine, Houston, TX USA; 3 Department of Genetics and Genomic Sciences, Icahn School of Medicine at Mount Sinai, New York, NY USA; 4 Masonic Cancer Center, University of Minnesota, Minneapolis, MN USA; 5 Department of Obstetrics, Gynecology, and Women’s Health, University of Minnesota, Minneapolis, MN USA; 6 University of Arkansas for Medical Sciences, Little Rock, AR USA

**Keywords:** Ovarian cancer, Predictive markers

## Abstract

Platinum-based chemotherapy, including cisplatin, carboplatin, and oxaliplatin, is prescribed to 10-20% of all cancer patients. Unfortunately, platinum resistance develops in a significant number of patients and is a determinant of clinical outcome. Extensive research has been conducted to understand and overcome platinum resistance, and mechanisms of resistance can be categorized into several broad biological processes, including (1) regulation of drug entry, exit, accumulation, sequestration, and detoxification, (2) enhanced repair and tolerance of platinum-induced DNA damage, (3) alterations in cell survival pathways, (4) alterations in pleiotropic processes and pathways, and (5) changes in the tumor microenvironment. As a resource to the cancer research community, we provide a comprehensive overview accompanied by a manually curated database of the >900 genes/proteins that have been associated with platinum resistance over the last 30 years of literature. The database is annotated with possible pathways through which the curated genes are related to platinum resistance, types of evidence, and hyperlinks to literature sources. The searchable, downloadable database is available online at http://ptrc-ddr.cptac-data-view.org.

## Introduction

Cisplatin, the first platinum-based anti-cancer therapy, was approved by the FDA for treating testicular cancer in 1978, approximately ten years after its cytotoxic function was accidentally discovered by Barnett Rosenberg [1]. In an effort to reduce side effects and increase cytotoxicity, two new platinum compounds, carboplatin and oxaliplatin, were subsequently developed and approved by the FDA. Other formulations have been approved in other countries and novel platinum formulations are currently being developed. It is estimated by the National Cancer Institute that approximately 10–20% of all cancer patients will receive a platinum drug during the course of their therapy (https://www.cancer.gov/research/progress/discovery/cisplatin).

**Fig. 1:**
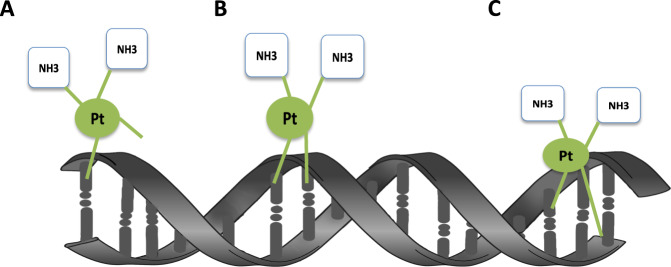
Platinum mechanism of action. Platinum cytotoxicity is mainly caused by formation of (**A**) DNA adducts and (**B**) intra- or (**C**) inter-strand crosslinks. The adducts and crosslinks inhibit DNA duplication leading to a DNA damage response and eventual cell death.

**Fig. 2:**
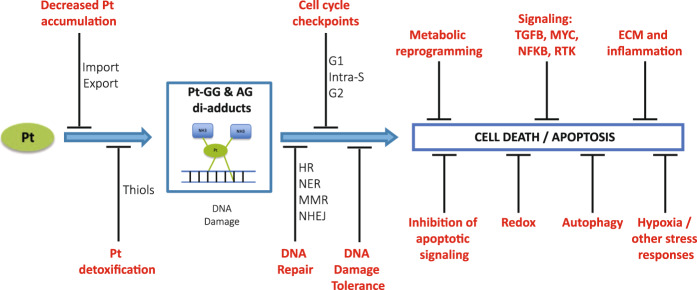
Mechanisms of platinum resistance. Tumors employ multiple mechanisms to resist platinum-induced cell death. These mechanisms can be categorized as following: (1) Reduced importation and increased exportation, sequestration, and detoxification of platinum (Pt); (2) Enhanced repair and tolerance of platinum induced DNA damage and blockage of cell cycle inhibition; (3) Inhibition of apoptotic signaling, downregulation of reactive oxygen species (ROS), and increased autophagy; (4) Hypoxia and other stress responses (e.g. ER stress response); (5) Metabolic reprogramming; (6) Upregulation of key signaling pathways promoting resistance; and (7) Extracellular mechanisms that alter the extracellular matrix (ECM) and enhance tumor-promoting inflammation.

The main cytotoxic mechanism of platinum is due to the generation of mono adducts and crosslinks in DNA, although other cytotoxic activities have been reported [2] (Fig. 1). The DNA damage caused by platinum results in severe cellular stress, apoptosis, and immune responses that collectively account for the anti-cancer effect of platinum [2]. Tumor response to platinum treatment is complex, heterogeneous, and involves both tumor-intrinsic and tumor-extrinsic activities. Perhaps correspondingly, the development of resistance encompasses multiple mechanisms. Extensive research has been conducted over the last three decades to understand these mechanisms of resistance, which can be broadly categorized into the following biological processes: (1) regulation of drug entry, exit, accumulation, sequestration, and detoxification, (2) enhanced repair and tolerance of platinum induced DNA damage, (3) alterations in cell survival pathways, (4) alterations in pleiotropic processes and pathways, and (5) changes in the tumor microenvironment (Fig. 2). Understanding the complex response to platinum-based therapy will be crucial for developing improved methods for overcoming platinum resistance.

Several excellent reviews have been published covering the topic of platinum resistance [2–4], although no comprehensive, annotated database of genes associated with platinum resistance has been reported. Due to the advent of large-scale genomics and proteomics, the development of annotated sets of functionally related genes/proteins has become increasingly valuable for performing sophisticated analyses, including the use of artificial intelligence. To facilitate a systems biology approach to platinum resistance, we conducted an updated literature review of >800 publications covering the last 30 years of research on platinum resistance and developed a highly curated and annotated database of the >900 genes/proteins implicated in platinum response (Supplementary Table S1). For each gene, we provide descriptions of the role the gene may play in platinum resistance, hyperlinks to citations that support this role, and a numerical annotation of the type of supporting evidence for each gene, as well as associated pathways. Additionally, we have created a data portal for browsing, searching, and downloading the gene sets and annotations. An overview of the categories of platinum resistance mechanisms represented in the database is provided below.

## Regulation of drug entry, exit, accumulation, sequestration, and detoxification

### Blocking entry

Cisplatin is actively transported into cells via the plasma membrane sodium pump, copper transporter, and organic cation transporters [5]. Reduced expression of genes encoding the protein constituents of these pumps (*SLC31A1/CTR1*, *SLC22A1/OCT1*, *SLC22A2/OCT2*, and *SLC22A3/OCT3*) can lead to platinum resistance (Supplementary Table S1). Endocytosis may be another mechanism of platinum importation, as decreased endocytosis has been associated with platinum resistance [6], while genes involved in regulating endocytosis (e.g., *RAB5C* or *TFRC*) are also associated with platinum resistance (Supplementary Table S1).

### Enhancing efflux

In addition to blocking entry, enhanced drug efflux can lead to cisplatin resistance. Two copper exporters, P-type ATPases encoded by *ATP7A* and *ATP7B*, modulate cisplatin export [5] and increased expression is associated with cisplatin-resistant tumor cells and poor clinical response in ovarian and endometrial cancer patients (Supplementary Table S1). Many studies have implicated members of the ATP-binding-cassette (ABC) family of transporters, especially the C sub-family consisting of the multidrug resistance-associated (MRP) genes, including *ABCB1/PGP*, *ABCC1/MRP1*, *ABCC2/MRP2*, *ABCC3/MRP3*, *ABCC4/MRP4*, and *ABCC5/MRP5* in platinum resistance (Supplementary Table S1). There is strong evidence that *ABCC2/MRP2* plays a major role in platinum therapy resistance, since platinum resistant cells have increased expression, and exogenous overexpression induces resistance while reduced expression re-sensitizes cells to platinum [7]. Clinically, high expression levels of both *ABCB1/PGP* and *ABCC2/MRP2* are associated with platinum resistance in cancer patients (Supplementary Table S1). In addition to increased expression, alterations in glycosylation of two ABC transporters, ABCC1/MRP1 and ABCC4/MRP4 (due to reduced levels of glycosylation enzymes *GNPTG* and *MGAT5*), result in platinum resistance [8]. Elevated levels of another transporter, MVP/LRP, also correlates with platinum resistance [9].

### Sequestration and detoxification

Cancer cells will actively sequester platinum drugs by generating nucleophilic scavengers, including glutathione (GSH) and metallothioneins, which avidly bind and inactivate platinum drugs. GSH is synthesized in a two-step process requiring glutamate cysteine ligase (encoded by *GCLC* and *GCLM*) and glutathione synthase (encoded by *GSS*) [10]. Once synthesized, the glutathione S-transferase (GST) family of enzymes will catalyze the binding of platinum to GSH [11]. Increased levels of GSH are significantly correlated with platinum resistance in several tumor models; increased levels of many of the enzymes, including *GCLC*, *GSTA1*, and *GSTP1*, are associated with platinum resistance in cell lines; and correlations between platinum resistance and elevated levels of *GSTP1* were found in cancer patient samples, which correlated with poor survival (Supplementary Table S1). In addition to sequestration, platinum-bound GSH is more readily exported by ABCC1/MRP1 and ABCC2/MRP2, contributing to enhanced resistance [12]. Similar to GSH, increased levels of metallothioneins (MTs), including *MT1A*, *MT2A*, and *MT3*, have been implicated in platinum resistance, and high levels of the major MT forms (MT1 and MT2) are correlated with reduced survival in patients receiving platinum-based therapy (Supplementary Table S1).

## Enhanced repair and tolerance of platinum induced DNA damage

The main cytotoxic effect of platinum is due to the formation of DNA adducts leading to a DNA damage response (DDR) that ultimately results in apoptosis. Cancer cells have developed multiple mechanisms to thwart this response by blocking the DDR at multiple levels.

### Reduced mismatch repair

Platinum-induced DNA adducts physically distort the DNA helix, and these distortions are detected by proteins including HMGB1/HMG1, HMGB2/HMG2, and the mismatch repair (MMR) proteins (including MSH2, MLH1, and MSH6) [13]. These proteins trigger the initiation of DDR, but are unable to remove the adduct, eventually leading to apoptosis. Reduced expression of *MLH1*, *MSH2*, and *MSH6* are all correlated with increased platinum resistance in cell lines and patients of some cancer types (Supplementary Table S1).

### Enhanced DNA repair

Platinum adducts result in DNA intrastrand and interstrand crosslinks (ICL), which result in activation of the DDR. ICL repair involves parts of nuclear excision repair (NER), homologous repair (HR), translesion synthesis (TLS), and Fanconi anemia (FA) proteins.

#### Nucleotide excision repair (NER)

Over 20 proteins are associated with NER, and inactivation of many of these will cause increased platinum sensitivity. Conversely, upregulation of a few rate-limiting proteins leads to platinum resistance [14]. For example, increased expression of the NER genes *ERCC1* and *ERCC4/XPF* is associated with cisplatin resistance in ovarian cancer cells, while knockdown of *ERCC1* enhanced cellular sensitivity to cisplatin [15]. Clinically, low expression or mutations of many NER genes including *ERCC1*, *ERCC2/XPD*, *ERCC3/XPB*, *ERCC4/XPF*, *ERCC5/XPG*, *ERCC6*, *ERCC8*, and *XPA* are prognostic for favorable response to platinum (Supplementary Table S1).

#### Fanconi anemia (FA)

FA is a recessive, cancer-prone disease associated with cellular sensitivity to DNA cross-linking agents such as platinum. Downregulation or mutation of genes in the FA pathway results in enhanced sensitivity to platinum drugs [16]. For example, downregulation of *FANCA*, *FANCF*, *FANCL*, or *FANCD2* potentiated cisplatin sensitivity in lung cancer cells (Supplementary Table S1). Defects in formation of *FANCD2* nuclear foci after cisplatin treatment can be used to indicate cisplatin sensitivity [17] and concomitant overexpression of *PARP1*, *FANCD2*, and *TP53* predicted increasing resistance to platinum [18].

#### Homologous recombination and non-homologous end joining (HR and NHEJ)

ICLs frequently lead to double strand breaks (DSBs), which require HR or NHEJ for repair. HR-defective (HRD) cancers are often more sensitive to platinum than their HR-proficient counterparts. Two critical components of the HR system are encoded by *BRCA1* and *BRCA2*. Loss of the wild-type allele of either of these two genes is frequently observed in familial breast and ovarian cancers [19] and *BRCA1/2*-deficient ovarian cancer patients have generally better responses to platinum and are associated with better clinical outcome [20]. Over time, however, the majority of these *BRCA1/2*-deficient cancers become resistant. In some cases, cisplatin resistance can develop due to secondary mutations that restore *BRCA1* or *BRCA2* functions [20]. In addition to *BRCA1/2*, other HR proteins also have important prognostic and predictive value for platinum-based therapy. For example, *RAD51* foci in the nuclei of cells after cisplatin exposure has been used as an indicator of HRD and platinum sensitivity [21], and high expression levels of *MRE11A* and *RAD50* are independent predictors of platinum resistance in gastric cancer [22]. Furthermore, defects in proteins that regulate HR can also cause HRD and platinum sensitivity. For example, CDK12 phosphorylates the C-terminal domain of RNA polymerase II, regulating transcription of a subset of HR genes including *BRCA1* [23]. Somatic *CDK12* mutations or chemicals that disable CDK12 function in ovarian cancer cells reduce *BRCA1* levels, disrupt HR repair, and sensitize these cells to platinum [24]. Unlike HR, NHEJ performs DSB repair without requiring a sister chromatid to serve as a template and may compete with HR during DSB repair [25]. Altered activities of NHEJ genes including *XRCC5/KU80*, *XRCC6/KU70*, *PRKDC/DNA-PKcs*, and *TP53BP1* can be either negatively or positively associated with platinum resistance, depending on types of cancer cell lines or clinical samples (Supplementary Table S1).

#### Translesion synthesis (TLS)

TLS enables cells to replicate DNA past the adduct to avoid replication fork collapse and death signaling. This increases the tolerance of tumor cells to high levels of platinum-induced DNA adducts and results in platinum resistance. On the other hand, because TLS can be error-prone, in addition to its role in cell survival upon cisplatin treatment, it also introduces genetic instability, heterogeneity, and platinum-resistant subpopulations [26]. The bypass of platinum adducts by TLS is mediated by a specific group of DNA polymerases including *POLH*, *POLI*, *POLK*, *REV1*, as well as *REV3L/POLZ* and *MAD2L2/REV7*, and increased expression of these genes is correlated with platinum resistance [14]. For example, a high level of *POLH* is associated with cisplatin resistance in lung and bladder cancer (Supplementary Table S1). *REV3L/POLZ* overexpression confers cisplatin resistance in a glioma cell line, while depletion of this protein diminishes cisplatin resistance (Supplementary Table S1). Clinically, *REV3L/POLZ* and *REV1* germ line mutations were found to be significantly associated with longer overall survival of malignant mesothelioma patients after platinum-based therapy (Supplementary Table S1).

Besides their role in HR, *BRCA1* and *BRCA2* also protect stalled replicative forks by preventing degradation of nascent DNA by *MRE11A* [27]. Knockdown of PAX-interacting protein 1 (*PAXIP1*) or nucleosome remodeling factor *CHD4* restored cisplatin resistance in *BRCA2*-deficient B lymphocytes and an ovarian cancer cell line via inhibition of recruitment of *MRE11A* and diminished degradation of replication forks, independent of HR [27, 28]. Moreover, *RADX* was found to antagonize the fork-stabilizing activity of *BRCA2* and *RAD51*. Like *PAXIP1* and *CHD4*, loss of *RADX* restores fork protection and cisplatin resistance to *BRCA2*-depleted cells without restoring HR [29]. On the other hand, homologous recombination OB-fold protein (*HROB*) interacts with RPA1 and MCM8/9 to facilitate HR and replication fork progression. Loss of *HROB* resulted in cisplatin sensitivity, slower replication fork progression, and increased fork stalling in response to cisplatin [30]. Consistently, *BRCA2* mutated ovarian cancers with reduced *PAXIP1* or *CHD4* expression are associated with shorter progression-free survival and shorter overall survival [27, 28]. Furthermore, in 3D organoid cultures derived from HGSOC patients, replication fork stability was found to correlate with carboplatin sensitivity [31]. Therefore, the status of replication fork stability may be useful for predicting responses to platinum.

#### Base excision repair (BER)

The relationship between BER and platinum resistance is complicated: (1) activity of *UNG*/*UNG1* and *POLB* is negatively associated with platinum resistance; (2) activity of *FEN1*, *LIG3*, and *XRCC1* are positively correlated with platinum resistance; and (3) *APEX1* and *MUTYH* are either positively or negatively associated with platinum resistance, depending on cell context (Supplementary Table S1). These BER proteins have multiple other functions related to platinum response in addition to their functions in BER, which could account for the discordant effects. Nevertheless, there are also reports of direct connection between BER and repair of platinum damage [32].

### Alterations to cell cycle regulators triggered by DNA damage

Maintaining replication competence in the face of DNA damage is a hallmark of cancer and a requirement for platinum resistance. Overcoming cell cycle checkpoints is accomplished via multiple mechanisms, with one of the most important being inactivation of the master cell cycle regulator, TP53. DNA damage results in activation of ATM and ATR, which results in stabilization of the TP53 protein causing increased levels of cell cycle inhibitors, including *CDKN1A/p21*^*Cip*^, *CDKN1B/p27*^*Kip1*^, and *CDKN1C/p57*^*Kip2*^ [33]. Initiation of cell cycle arrest can have contradictory effects on cells depending on context, resulting in either protection or senescence/apoptosis. Correspondingly, the cellular response to changes in the level of checkpoint inhibitors is complex. For example, both increased and decreased levels of p21^Cip^ have been associated with increased platinum resistance [34]. Similar effects are seen with another major cell cycle inhibitor *CDKN2A/p16*^*INK4a*^, where decreased levels can result in platinum resistance, while in other cases, result in platinum sensitivity (Supplementary Table S1). Pharmacologic reversal of platinum resistance has been achieved in some preclinical models using inhibitors of the cell cycle regulators *CHEK1* and *CHEK2* (Supplementary Table S1).

## Alterations in cell survival pathways

### Blocking apoptosis

Both pro-survival and apoptotic signaling pathways are triggered at the same time upon platinum exposure. The final fate of the cell is dependent on the relative intensity and duration of these opposing signals. MAPK pathways, including ERK, p38, and JNK are generally pro-survival, and increased activation of these pathways is generally correlated with increased platinum resistance [35]. Exceptions are numerous, however, due to the pleiotropic effects of MAPK signaling. For example, comparison of a few cisplatin-sensitive cell lines and their cisplatin resistant derivatives revealed that p38 and JNK failed to be activated in a sustained fashion in response to cisplatin in the resistant cells [36, 37].

*TP53* is a master guardian of the genome and can trigger pro-survival or pro-apoptotic signals depending on cellular context. In most clinical settings, platinum resistance is associated with defective *TP53* functions. For example, in testicular germ cell tumors, in which *TP53* is predominantly wild type, the patients experience better cure rates to a cisplatin-based regimen than most other adult solid tumor patients [38]. However, in cell lines, the association of cisplatin resistance with mutant *TP53* is not clear or even negative. For example, cisplatin resistance sometimes correlates with the presence of wild type *TP53* [4]. Moreover, disruption of *TP53* function can increase cisplatin sensitivity in some cell lines [39].

Platinum generally triggers apoptosis via the intrinsic pathway initiated at the level of the mitochondria via induction of *BAX* and *BAK* through *TP53*-dependent or MAPK-dependent pathways [40]. Both proapoptotic and antiapoptotic Bcl-2 family members are associated with platinum resistance. For instance, reduced expression or inhibition of the proapoptotic proteins *BAD* and *BAX* is associated with poor prognosis in ovarian cancer patients receiving platinum-based chemotherapy (Supplementary Table S1). Overexpression of antiapoptotic proteins such as *BCL2*, *BCL2L1/BCL-XL*, and *MCL1*, on the other hand, negatively correlates with response to cisplatin in ovarian or head and neck squamous cell carcinoma (HNSCC) patients (Supplementary Table S1). In addition to Bcl-2, increased levels of members of the inhibitors of apoptosis (IAP) family including *BIRC2*, *BIRC3*, *BIRC5/survivin*, and *XIAP* are associated with cisplatin resistance and worse clinical outcome in patients of ovarian, esophageal, or head and neck cancer (Supplementary Table S1). Consistent with these findings, *XAF1*, an inhibitor of XIAP, is associated with a better survival rate in advanced bladder cancer and ovarian cancer patients treated with neoadjuvant chemotherapy (Supplementary Table S1).

In addition to triggering the intrinsic apoptotic pathway, platinum can induce apoptosis through the extrinsic pathway via activation of *FAS/FASLG* or the DR/caspase-8/caspase-3 pathway [4]. Some components in this pathway are also found to be associated with cisplatin sensitivity in clinical studies. For example, non-small cell lung cancer (NSCLC) patients with higher *TNFRSF10B/DR5* expression showed higher response rate to gemcitabine and cisplatin-based chemotherapy [41].

### Regulation of reactive oxygen species

In healthy cells, reactive oxygen species (ROS) are formed during the process of aerobic metabolism and are maintained at low levels. In cancer cells, oxidative stress leads to elevated ROS levels. Platinum therapy increases ROS levels, mostly via the mitochondria [42]. Several mechanisms for platinum-induced ROS have been delineated, including depletion of GSH [2], inhibition of *TXNRD1/TrxR1* [43], and other mechanisms involving cytochrome P450 and *MNSOD* [44, 45]. Normally, high levels of ROS causes DNA damage, organelle fragmentation, disruption of mitochondria function and depletion of ATP. The cellular response to elevated ROS is complex and involves activated MAPK and TP53 signaling [46]. Cancer cells escape cell death and damage induced by high ROS levels by increasing the production of NADPH and GSH, antioxidant molecules readily used by several ROS-scavenging enzymes to lower ROS levels, such as superoxide dismutases (SODs), catalase (CAT), and glutathione peroxidases (*GPX3*, *GPX4*) [47].

#### NADPH

Elevated levels of *G6PD* and *PGD*, the Pentose Phosphate Pathway (PPP) enzymes required for NADPH production, are present in cisplatin-resistant ovarian cancer cell lines, and knockdown of either gene sensitizes cisplatin-resistant cells (Supplementary Table S1). Moreover, ovarian and lung cancer patients with higher *PGD* levels have worse survival outcomes relative to patients with lower *PGD* expression (Supplementary Table S1). The transcription factor *NFE2L2/NRF2* is a key regulator of oxidative stress and regulates most NADPH-generating enzymes and redox proteins [48]. Upregulation of *NFE2L2/NRF2* or downregulation of *KEAP1*, an E3 ubiquitin ligase that tags NFE2L2/NRF2 for destruction, correlates with platinum resistance in advanced NSCLC (Supplementary Table S1).

#### GSH

In addition to the role of GSH in platinum detoxification via sequestration and efflux, GSH acts as an antioxidant to neutralize platinum-induced ROS, and high GSH levels are correlated with platinum resistance. Besides the rate-limiting enzyme *GCLC*, the availability of cysteine, glutamate, and glycine are also important determinants of GSH [49]. Reduced levels of the proteins required to generate these metabolic precursors of GSH, including *PHGDH*, *CBS*, *SLC7A11/xCT*, *GLS*, *GLS2*, and *SLC1A5/ACST2*, have been associated with increased platinum sensitivity (Supplementary Table S1).

#### Aldehydes

Platinum-induced ROS will generate cytotoxic levels of aldehydes via oxidative stress-induced lipid peroxidation [50]. High expression levels of enzymes involved in removing aldehydes, including Aldo-keto reductases (*AKR1B10*, *AKR1C1-3*) and Aldehyde dehydrogenases (*ALDH1A1*, *ALDH3A1*), were found to be associated with cisplatin resistance in various types of cancer patients including gastrointestinal, ovarian, bladder, NSCLC, and cervical cancer (Supplementary Table S1).

### Regulation of autophagy

Autophagy is a catabolic pathway that involves the sequestration and degradation of damaged organelles and recycles essential components in response to stress conditions, including cisplatin-induced stresses such as DNA damage, oxidative stress, and ER stress [51]. Autophagy also downregulates proapoptotic proteins and enhances DNA repair [52]. Thus, autophagy reduces stress signaling, maintains cellular homeostasis, and promotes survival upon platinum exposure.

Upregulation of the autophagic pathway has been shown to correlate with platinum resistance in lung cancer cells [53], and genes involved in production of autophagosomes, including *ATG5*, *ATG7*, *ATG12*, *ATG14*, and *BECN1*, promote platinum resistance (Supplementary Table S1). Similarly, elevated expression of *MAP1LC3A/LC3A*, a protein required for autophagosome membranes, is associated with platinum resistance in ovarian cancer [54].

## Alterations in pleiotropic processes and pathways

### Hypoxia and the endoplasmic reticulum stress response

#### Hypoxia

Cells in solid tumors are exposed to hypoxic conditions, and extensive hypoxia is correlated with platinum resistance and tumor progression [55]. Hypoxic transcriptional programs are mainly activated by the HIF transcription factors but can also be activated by other stress signaling pathways. The response is complex, as HIFs can regulate the transcription of up to 2% of the human genome [56]. The transcriptional response can result in apoptosis or cell survival, depending on cellular context. *HIF1A* contributes to platinum resistance indirectly through regulation of the expression of platinum resistance related genes: such as signaling genes *MMP9*, *CXCR4*, *CXCL8*, *TGFB3/TGF-β3*, and *VEGFA/VEGF*; DNA repair enzymes *PRKDC/DNA-PK*, *XRCC5/KU70*, *XRCC6/KU80*, *XPA*, *XPC*, and *XPD*; the drug efflux transporters *ABCC1/MRP1*, *ABCG2/ BCRP*, and *MVP/LRP*; and EMT transcription factors *TWIST1*, *ZEB1*, *ZEB2*, and *TCF3* [56–61] (Supplementary Table S1). *HIF1A* overexpression is associated with malignancy and poor prognosis in esophageal or head and neck squamous cell carcinoma patients under platinum-based chemotherapy (Supplementary Table S1). Additionally, loss of the E3 ubiquitin ligase (*VHL*) that tags HIFs for degradation is linked to platinum resistance in renal cancer (Supplementary Table S1).

#### Endoplasmic reticulum stress and the unfolded protein response

Platinum therapy results in endoplasmic reticulum (ER) stress, which triggers the unfolded protein response (UPR) due to the accumulation of misfolded proteins and disturbances in redox regulation [62]. To maintain cell viability, cancer cells must respond by altered regulation of genes and proteins that participate in this stress response. Heat shock proteins (HSP) are molecular chaperones induced during ER stress and the UPR [63]. Numerous studies have found that increased activities of the *HSPB1/HSP27*, *HSPD1/HSP60*, as well as HSP70, and HSP90 families of HSPs (*HSPA1A*, *HSPA5/GRP78*, *HSP90AA1*, *HSP90AB1*) are positively correlated with platinum resistance (Supplementary Table S1).

### Metabolic reprogramming

Metabolic reprogramming is an established hallmark of cancer. The Warburg effect was first discovered as a feature of metabolic reprogramming in cancer cells, whereby cancer cells have increased glucose uptake, hyperactivated glycolysis, decreased oxidative phosphorylation (OXPHOS), and the accumulation of lactate, even in the presence of abundant oxygen and functioning mitochondria [64]. Although increased aerobic glycolysis is considered a metabolic hallmark of tumors, its causal relationship with platinum resistance is still controversial. Platinum-resistant tumor cells can either prefer aerobic glycolysis (Warburg-like) or oxidative phosphorylation (OXPHOS-addicted). There are plausible explanations for either metabolic phenotype to promote tumorigenesis and the development of platinum resistance [65, 66].

There are reports supporting the role of enhanced aerobic glycolysis in promoting platinum resistance [67]. Conversely, inhibition of aerobic glycolysis can severely deplete ATP and restore platinum sensitivity in cancer cells using aerobic glycolysis [68]. Elevated expression of several key glycolytic enzymes, including *SLC2A1/GLUT1*, *HK2*, *ENO1*, *PGK1*, *PDK1*, *PDK4*, and *PFKFB3*, was found to correlate with resistance in some cancer cell lines and clinical tissue samples [69] (see also Supplementary Table S1).

On the other hand, the expression levels of proteins related to mitochondrial biogenesis and mitochondrial respiratory chain were found to correlate with platinum resistance in high-OXPHOS cancer cell lines, and inhibition of OXPHOS reduced cisplatin-resistance in these cells [70, 71].

AMPK is a major regulator of metabolic reprogramming, and its relationship to platinum resistance is also complex. There are reports that activation of AMPK enhances the cytotoxic effects of platinum drugs on cancer cells, but there are also opposing reports that low AMPK activities correlate with better outcomes in resected gastric cancer patients treated with cisplatin-based chemotherapy [72, 73].

### Translation regulation

Many cisplatin resistant cell lines express elevated levels of translation factors and ribosomal proteins [74], and many proteins associated with platinum resistance are encoded by mRNAs with complex structures and are highly sensitive to levels of the translation initiation factor EIF4E [75]. Excessive expression of *EIF4E* is associated with platinum resistance, while knockdown of *EIF4E* enhances the cytotoxic effects of cisplatin in cell lines and xenograft models [76]. EIF4E is activated by phosphorylation via the AURKA kinase, and overexpression of AURKA correlates with platinum resistance in high grade serous ovarian cancer (HGSOC) patients (Supplementary Table S1).

The mTOR pathway is a central regulator of protein production and can activate EIF4E by phosphorylating the inhibitory protein *EIF4EBP1* and by activating *RPS6KB1/p70S6K* [77]. Activation of this pathway is strongly linked to platinum resistance [78], and inhibition of *MTOR* enhanced cisplatin-induced apoptosis [79]. Similarly, the levels of phosphorylated *RPS6KB1/p70S6K* were elevated in the small cell lung cancer cells that acquired resistance to cisplatin, and activation of *RPS6KB1/p70S6K* contributes to cisplatin resistance (Supplementary Table S1).

On the other hand, some proteins that downregulate mRNA translation may exert inhibitory effects on the growth and proliferation of tumors and enhance sensitivity to platinum compounds. For example, PDCD4 can suppress protein translation by directly interacting with EIF4A and EIF4G to inhibit the formation of the translation–initiation complex [80]. Overexpression of *PDCD4* enhances platinum sensitivity, while knockdown of *PDCD4* reduces platinum sensitivity in ovarian cells and in a xenograft model [81].

*EIF3A*, the largest subunit of the eIF3 complex, has peak expression at S-phase and was reported to downregulate the translation of NER proteins *XPA*, *XPC*, *RAD23B*, and *RPA2* [82]. Consistent with the role of NER in platinum sensitivity (discussed above), knockdown of *EIF3A* in nasopharyngeal and ovarian carcinoma cell lines increases cellular resistance to cisplatin. *EIF3A* expression levels also correlate with better response to platinum-based chemotherapy in lung and ovarian cancer patients (Supplementary Table S1).

### Epigenetic alterations

Epigenetic changes contribute to platinum resistance. For example, elevated expression of *SMARCA4*, a member of the SWI/SNF chromatin remodeling complex, promotes cisplatin resistance in pancreatic ductal epithelial cells [83], and low expression predicts responsiveness to platinum therapy in NSCLC [84]. Histone H3 acetylation enhances *CD274/PD-L1* expression, which contributes to chemoresistance [85]. High expression of *CD274/PD-L1* combined with low expression of the histone deacetylase, *HDAC3*, correlates with platinum resistance in NSCLC [85]. Histone methylation, on the other hand, can either enhance or suppress transcription depending on the context. Knockdown of the histone methyltransferase *KMT2B* reverses platinum sensitivity of *BRCA1* or *BRCA2* deficient cancer cells by stabilizing replication forks [27], while elevated levels of EZH2, another histone methyltransferase, correlates with poor response to platinum therapy [86].

DNA methylation is generally associated with suppression of transcription, and hypermethylation resulting from aberrant DNA methyltransferase *DNMT1* activity has been associated with tumor suppressor gene silencing and chemoresistance in ovarian and other cancer types (Supplementary Table S1). Examples of DNA methylation affecting platinum sensitivity include hypermethylation of the MMR gene *MLH1* promoter correlating with platinum resistance in cell lines and predicting poor patient survival [87, 88], and methylation of *BRCA1* correlating with platinum sensitivity/response in breast cancer [89].

### Alterations in major signaling pathways

#### TGFβ signaling

In general, tumors co-opt TGFβ signaling to induce metastasis and chemoresistance through promoting angiogenesis, EMT, increased GSH production, a stem cell phenotype, ECM remodeling, and immune escape [90]. Elevated levels of TGFβ at tumor sites correlate with poor prognosis and treatment resistance in human patients with cancer [91, 92].

TGFβ is a potent inducer of EMT, which is strongly associated with platinum resistance [93]. Moreover, a gene expression signature associated with TGFβ-activated EMT has been identified in platinum resistant ovarian cancer tissues and can predict platinum resistance [94]. The underlying mechanisms of how TGFβ-activated EMT supports chemoresistance in cancer cells has not been clearly elucidated, but are likely due to increased expression of efflux pumps, DNA repair genes, and effects on major signaling pathways including AKT and TP53 [93, 95]. Many studies have demonstrated that downregulation of EMT-inducing transcription factors including *TWIST1*, *SNAI1/SNAIL*, *SNAI2/SLUG*, and *ZEB1/2* leads to platinum sensitivity, while upregulation leads to platinum resistance (Supplementary Table S1).

TGFβ signaling also generates cancer stem cell (CSC) phenotypes, which are linked to cancer progression and treatment resistance. Several studies support the hypothesis that CSCs are responsible for chemoresistance and tumor relapse after conventional chemotherapy removes the bulk population of non-CSCs [96, 97]. Multiple markers of the CSC phenotype (e.g., *CD44*, *CD24*, *ALDH1A1*, *NANOG*) are found to be correlated with platinum resistance in cancer patients of various types (Supplementary Table S1).

#### MYC signaling

In human ovarian tumors, amplification of *MYC* is common [98, 99], and high expression of *MYC* is associated with platinum resistance in ovarian cancer [100]. *MYC* promotes platinum resistance mainly through transactivation of a broad range of genes that drive glucose metabolism, glutamine metabolism, one carbon metabolism, fatty acid synthesis, oxidative phosphorylation, nucleotide synthesis, and protein biogenesis, which results in increased cell survival and proliferation [101]. For example, *MYC* promotes glutamine import by directly inducing the expression of key glutamine transporters *SLC7A5*, *SLC1A5*, and *GLS* [102] and it is well documented that increased utilization of glutamine induces platinum resistance [103]. The mechanism is not entirely clear, but likely includes increased nucleotide and glutathione synthesis [49, 103]. Myc signaling also results in protecting cells from platinum-induced ROS toxicity by activating a mitochondrial methyl transferase (*SHMT2*), which results in elevated NADPH [104], and by activating *NFE2L2/NRF*, a master transcription factor regulating ROS-detoxifying enzymes [48].

#### NFκB signaling

Another well-known transcription factor complex that promotes cancer cell survival is nuclear factor kappa-light-chain-enhancer of activated B cells (NFκB), a family of transcription factors constituted by dimers of different combinations of 5 proteins sharing structural homology (*NFKB1*, *NFKB2*, *RELA*, *RELB*, and *REL*) [105]. Higher levels of NFκB correlate with platinum resistance and poor outcome in ovarian cancer [106]. Interestingly, cisplatin itself likely causes upregulation of NFκB via activation of the DDR, resulting in ATM-mediated phosphorylation of the NFκB inhibitors, *CHUK/IKKα* and *IKBKB/IKKβ* [107]. Mechanistically, NFκB promotes survival by activating anti-apoptotic genes, including *TRAF1*, *TRAF2*, *BIRC2/cIAP1*, BIRC3/cIAP2, *XIAP*, *BCL2A1/Blf/A1*, *BCL2L1/Bcl-XL*, and *CFLAR/FLIP* [108, 109], as well as detoxifying and redox genes such as *NQO1*, *SOD1*, and *SOD2* [110]. NFκB also triggers inflammatory responses via induction of various pro-inflammatory genes, including those encoding cytokines [111], which are associated with platinum resistance (see Section 5b).

#### Receptor tyrosine kinase signaling

Receptor tyrosine kinase (RTK) signaling triggers cell growth and blocks apoptosis, enhancing cancer cell survival and proliferation in the presence of platinum and other anti-cancer drugs [112, 113]. Many studies have specifically implicated the PI3K/Akt and the Ras/MAPK signaling pathways in the induction of platinum resistance. For example, overexpression of the EGFR family member *ERBB2/HER2/NEU*, which activates the PI3K/Akt pathway, is associated with platinum resistance in ovarian, NSCLC, and HNSCC cancer patients (Supplementary Table S1). In support of this observation, pharmacological targeting of MTOR, a key downstream signaling kinase in the pathway, can re-sensitize tumors to platinum drugs [78, 79]. Both the PI3K/Akt and Ras/MAPK signaling will phosphorylate the proapoptotic protein BAD, causing inactivation due to cytoplasmic sequestration, which leads to enhanced platinum resistance by blocking apoptosis [114]. Similarly, both AKT and MAPK signaling result in activation of the AP1 transcription factor complex (*FOS* and *JUN*), and overexpression of these genes are associated with platinum resistance, while downregulation re-sensitizes tumor cells to platinum [115] (see also Supplementary Table S1).

Activation of the Ras/MAPK pathway via activating mutations in RAS or overexpression of RAS confers resistance to platinum [115], while downstream of RAS, high expression of MAP2K1/MEK1 is associated with platinum resistance and correlates with reduced progression free survival of patients [116].

## Changes in the tumor microenvironment

### Extracellular matrix remodeling

The effects of the extracellular matrix (ECM) on cells are primarily mediated by integrins, a family of cell surface receptors that attach cells to the matrix and trigger signal transduction [117]. Alterations in the ECM can initiate integrin signaling and confer platinum resistance to cancer cells. For example, collagen and fibronectin in the ECM can synergistically activate PI3K/Akt signaling, causing increased cancer cell resistance to cisplatin. Increased levels of one collagen variant, *COL1A1*, enhances cisplatin resistance in liver and ovarian cancer cells via upregulation of MAPK and mTOR pathways (Supplementary Table S1). Another variant, *COL11A1*, activates PI3K/Akt and NFκB pathways to exert anti-apoptotic effects that are associated with chemoresistance [118]; high expression of this variant was significantly associated with platinum resistance and clinical outcome in ovarian cancer (Supplementary Table S1). On the other hand, Akt signaling can be activated by upregulation of fibronectin on stroma cells and contributes to platinum resistance in ovarian cancer cells [119], and high levels of FN1 in serum have positive correlation with recurrence and shorter progression free survival in ovarian cancer patients under platinum-based therapy [120]. Some of the integrins responsible for these resistance signals have been identified, including *ITGA5*, *ITGA6*, *ITGB1*, and *ITGB6*, where elevated levels are correlated with reduced overall survival and cisplatin resistance in human hilar cholangiocarcinoma patients and ovarian cancer (Supplementary Table S1). Downstream of integrin signaling, the focal adhesion kinase PTK2/FAK, was also found to drive platinum resistance by promoting survival [121]. Changes in the ECM are instigated by both cancer cells and cancer-associated fibroblasts. For example, ovarian cancer associated stroma cells are able to induce platinum resistance via induction of the fibronectin/Akt signaling pathway [119].

### Immune system induced inflammation

Tumor-promoting chronic inflammation leads to an innate immune response that promotes cancer development and progression and can contribute to platinum resistance [122]. Activation of the innate immune response results in the production of cytokines, including interleukins, interferons and tumor necrosis factor, which have all been associated with platinum resistance [123]. Nonetheless, the effects of cytokines are complex and often context-dependent, in some cases causing increased platinum resistance, while in other cases sensitizing tumors to platinum therapy.

Multiple lines of evidence indicate that *TNF*/*TNFA* expression enhances cisplatin resistance in cell lines, and elevated expression is associated with poor patient outcome (Supplementary Table S1). Similarly, high expression levels of many interleukins including *IL6*, *IL7*, *CXCL8/IL8*, *IL11*, and *IL17A/IL17* are correlated with increased platinum resistance or poor clinical outcome in several types of cancer (Supplementary Table S1). The JAK/STAT pathway, commonly activated by these cytokines, stimulates STAT transcription factors including *STAT1* and *STAT3* [124]. While *STAT1* activity could indicate either platinum response or resistance, high levels of *STAT3* is generally associated with platinum resistance (Supplementary Table S1). Plausible mechanisms for *STAT3*-enhanced resistance include upregulation of the anti-apoptotic proteins BCL2 and BIRC5/Survivin, the oncogene *MYC*, EMT genes [125], and activation of ATF6, resulting in ER stress response and autophagy [126].

On the other hand, when *IFNG*/Interferon-γ is used as a treatment, it can enhance the therapeutic effect of cisplatin in ovarian cancer, as shown by reduced tumor volume and prolonged progression-free survival [127]. Supporting the positive effect of *IFNG*, induction of the IFNG/STAT1 pathway correlates with improved treatment response in ER negative breast cancer in a mouse model [128]. Moreover, contrary to the harmful effects of TNF cited above, when TNF is targeted to cancer endothelial cells in refractory NSCLC patients, there has been evidence of enhanced cisplatin toxicity [129]. Similarly, administration of *IL1A*, *IL7*, or *IL24* enhanced the anti-tumor efficacy of cisplatin in lung cancer (Supplementary Table S1).

Other platinum resistance mechanisms induced by the innate immune response include upregulation of the AP1 oncogenes (*FOS* and *JUN*) [130], upregulation of pro-survival genes including *HSP70* and *HMOX1/HO-1* [131, 132], increased drug efflux [133], and activation of the EMT program [97].

## Database of genes associated with platinum resistance

The accompanying gene table (Supplementary Table S1) was generated following an extensive literature search. Publications reporting on platinum resistance were identified in Pubmed (The MEDLINE database) using the python NLTK package with the following search keywords: (“platinum” OR “cisplatin”) AND (“resistance” OR “sensitivity”) AND “cancer” AND (Date of publication: 1988–2020) along with all human gene symbols. Inclusion was also manually curated based on the expertise of our group. Publications were selected only if they contained genes that (1) showed differential expression between platinum resistant and sensitive cancer cells (or tissues); and (2) whose gene knockdown, mutation, or overexpression resulted in changes in platinum sensitivity. Our final database contains 1,628 unique PMID citations.

**Fig. 3:**
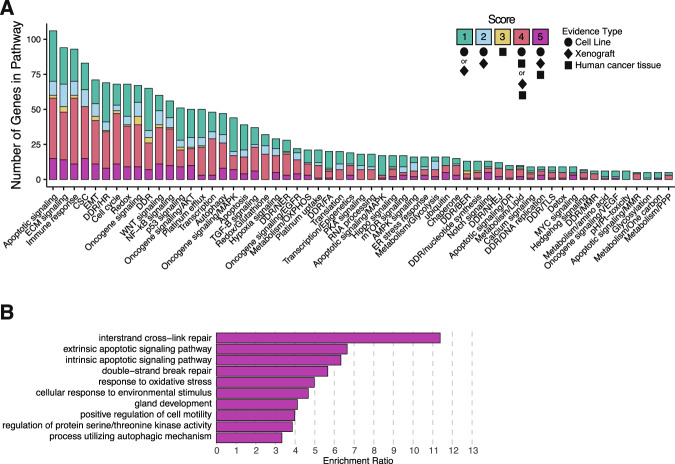
Pathway annotation of genes. **A** Number of genes (in Supplementary Table S1) at each level of evidence assigned to each putative platinum resistance mechanism pathway. Genes are assigned evidence scores based on experiments from cell lines, xenograft models, and/or human cancer tissue. Only pathways with at least five genes are shown. **B** Top GO biological process term enrichment of genes in Supplementary Table S1.

Our database includes the following information from the literature review: gene and protein identifiers including the NCBI Gene ID, Uniprot protein ID, HUGO gene symbol, and alternate IDs; manually curated functional annotations related to platinum resistance based on the literature review, with hyperlinked citations; and manually curated annotations indicating whether gene expression is increased or decreased during development of platinum resistance and pathways associated with the gene. We added a score annotation describing the strength of evidence supporting the gene’s role in platinum resistance (Supplementary Table S2, Fig. 3A). This score may be used as a threshold to filter the table based on the level of evidence (cell line, animal model, and/or human tissue). To complement our manual pathway annotation, we performed an over-representation analysis to identify enriched Gene Ontology (GO) Biological Processes using WebGestalt [134]. Top enriched functions included apoptosis, oxidative stress, and DNA repair (Fig. 3B, full results in Supplementary Table S3). Finally, targets of FDA approved drugs were annotated [135].

A searchable, filterable, and downloadable version of the table is available online at http://ptrc-ddr.cptac-data-view.org. This web application includes an interactive visualization of the number of genes assigned to each putative platinum resistance mechanism pathway.

## Concluding remarks

Based on 30 years of literature, multiple mechanisms are exploited by cancer cells to evade the cytotoxic effects of platinum therapy. Experimental evidence from the platinum-resistant PEO4 ovarian cancer cell line serves as a striking example of the multifactorial nature of platinum resistance. Multiple mechanisms of platinum resistance have been implicated in PEO4, including: a) restoration of DDR via a *BRCA2* reversion mutation [136]; b) alterations in histone methylation via overexpression of Histone-lysine N-methyltransferase *EZH2* [86]; c) activation of *HIF1A* [137]; d) activation of TGFβ signaling [138]; e) inhibition of pro-apoptotic Bcl2 family proteins [139]; and f) activation of transcription factor *STAT1* by deacetylase *HDAC4* [140]. Clonal heterogeneity within tumors exacerbates this phenomenon, as platinum resistance is often driven by pre-existing resistant subclones co‑inhabiting a single tumor mass and interspersed with sensitive cancer cell clones [141]. This multifaceted nature of resistance poses a major hurdle for developing effective therapeutic approaches targeting platinum resistant tumors, as well as predictive biomarkers of platinum response. Identifying and targeting dominant signaling pathways and networks of proteins that drive resistance will be important. One promising approach is to use the rapidly expanding omics datasets created by next generation technologies. To effectively interpret these large datasets, it will be necessary to develop new bioinformatic algorithms using systems biology and artificial intelligence. The gene list and gene matrix transposed (GMT) files, as well as the online data portal, provided with this review can be utilized to add knowledge-based annotations to these datasets.

Further confounding our ability to therapeutically target mechanisms of platinum resistance is that specific mechanisms can be context dependent. For example, upregulation of *MYC* promotes platinum resistance through activation of a broad range of genes that promote proliferation, redox balance, and energy generation. However, *MYC* is also implicated in platinum-induced p53 mediated apoptosis [142]. While high expression of *MYC* is associated with platinum resistance in *TP53* mutant ovarian cancer [86, 100], the opposite is seen in ER-positive breast cancer, where high expression is associated with platinum sensitivity [143]. The cell cycle inhibitor, *CHEK2*, serves as another example. In some ovarian cancer cell lines, the cell cycle checkpoint and DDR functions of *CHEK2* causes increased resistance to cisplatin [144]. However, in esophageal squamous cell carcinomas and some ovarian cancers, higher expression of *CHEK2* is associated with favorable response to cisplatin-based therapy [145, 146]. The complex response to oxidative damage induced by platinum therapy normally results in elevated ROS levels, and cancer cells with lower ROS levels are generally less susceptible to platinum-induced oxidative damage [47] and, therefore, are associated with platinum resistance. In these tumor cells, glycolytic metabolism could support the energetic demand that allows cellular proliferation and tumor expansion [64], while downregulation of OXPHOS diminishes ROS production and reduces the threshold for apoptosis induced by platinum [147]. However, in other platinum-resistant cancer cells, which often have increased endogenous oxygen consumption and mitochondrial activity, higher sublethal ROS levels have direct causative effects on platinum resistance through activation of antioxidation system and proliferative signaling [43, 133]. For example, increased expression of the antioxidant thioredoxin-1 (*TXN*) is associated with lower ROS and decreased cisplatin-resistance in a lung cancer cell line [43]. In contrast, upregulation of *TXN* is associated with an increased resistance to cisplatin in other cell lines [148, 149]. These results demonstrate the importance of cellular context and will need to be considered when developing therapies targeting platinum resistance mechanisms.

Platinum drugs are effective anticancer drugs used for treatment of epithelial neoplasms. Unfortunately, in a large number of cases, tumors are able to subvert the cytotoxic effects of platinum, resulting in platinum resistance and cancer mortality. Here, we have reviewed the evidence supporting the hypothesis that a large number of genes can contribute to platinum resistance either directly or indirectly. We also provide a manually annotated database of these genes that can be incorporated into large-scale omics analyses to facilitate development of therapies aimed at overcoming platinum resistance.

## Supplementary information

Table S1

Table S2

Table S3

pt_resistance_mechanisms.gmt
